# Glycosphingolipid GM3 is localized in both exoplasmic and cytoplasmic leaflets of *Plasmodium falciparum* malaria parasite plasma membrane

**DOI:** 10.1038/s41598-021-94037-3

**Published:** 2021-07-21

**Authors:** Shiomi Koudatsu, Tatsunori Masatani, Rikako Konishi, Masahito Asada, Hassan Hakimi, Yuna Kurokawa, Kanna Tomioku, Osamu Kaneko, Akikazu Fujita

**Affiliations:** 1 Department of Molecular and Cell Biology and Biochemistry, Basic Veterinary Science, Faculty of Veterinary Medicine, Kagoshima University, Korimoto 1-21-24, Kagoshima, 890-0065 Japan; 2 Transboundary Animal Diseases Research Center, Joint Faculty of Veterinary Medicine, Kagoshima University, Korimoto 1-21-24, Kagoshima, 890-0065 Japan; 3 Department of Protozoology, Institute of Tropical Medicine (NEKKEN), Nagasaki University, Sakamoto 1-12-4, Nagasaki, 852-8523 Japan; 4 Present Address: Laboratory of Zoonotic Diseases, Faculty of Applied Biological Sciences, Gifu University, 1-1 Yanagido, Gifu, 501-1193 Japan; 5 Present Address: National Research Center for Protozoan Diseases, Obihiro University of Agriculture and Veterinary Medicine, Inada-cho, Obihiro, 080-8555 Japan

**Keywords:** Cell biology, Cell signalling, Lipid signalling

## Abstract

Lipid rafts, sterol-rich and sphingolipid-rich microdomains on the plasma membrane are important in processes like cell signaling, adhesion, and protein and lipid transport. The virulence of many eukaryotic parasites is related to raft microdomains on the cell membrane. In the malaria parasite *Plasmodium falciparum*, glycosylphosphatidylinositol-anchored proteins, which are important for invasion and are possible targets for vaccine development, are localized in the raft. However, rafts are poorly understood. We used quick-freezing and freeze-fracture immuno-electron microscopy to examine the localization of monosialotetrahexosylganglioside (GM1) and monosialodihexosylganglioside (GM3), putative raft microdomain components in *P. falciparum* and infected erythrocytes. This method immobilizes molecules in situ, minimizing artifacts. GM3 was localized in the exoplasmic (EF) and cytoplasmic leaflets (PF) of the parasite and the parasitophorous vacuole (PV) membranes, but solely in the EF of the infected erythrocyte membrane, as in the case for uninfected erythrocytes. Phosphatidylinositol 4,5-bisphosphate (PtdIns(4,5)P_2_) was localized solely in the PF of erythrocyte, parasite, and PV membranes. This is the first time that GM3, the major component of raft microdomains, was found in the PF of a biological membrane. The unique localization of raft microdomains may be due to *P. falciparum* lipid metabolism and its unique biological processes, like protein transport from the parasite to infected erythrocytes.

## Introduction

Lipid rafts are membrane microdomains exhibiting heterogeneity in the lipid bilayers of cellular membranes^1^. They are presumably present in all cells and can be biochemically isolated as detergent-resistant membranes (DRMs) because they are insoluble in cold, non-ionic detergents^2^. However, analysis by atomic force microscopy showed that membrane microdomains became into much larger DRMs during Triton X-100 treatment^3^. Therefore, biochemical DRM preparation, which was once assumed to represent rafts in situ, is now generally thought to be a collection of raft-philic molecules that are induced artificially by the experimental procedure^4^. To understand the raft microdomain in cellular membranes, it is important to determine the molecular composition of the cell membrane in situ at the nanometer scale.

Previously, we demonstrated that immuno-electron microscopy (EM) of freeze-fracture replicas could capture glycosphingolipids, which are the most abundant raft molecules in the plasma membrane, and objectively allow the analysis of their distribution at the submicrometer scale^5–7^. Our method immobilizes the membrane lipids using rapid freezing and vacuum evaporation of a thin metal (carbon/platinum) layer^8^. This physical fixing method has advantages over chemical fixation in that it rapidly freezes molecular movement (within ~ 0.1 ms; J. Heuser, personal communication) and can continually hold proteins and lipids in the replica carbon/platinum thin membrane. With this technique, we found that two glycosphingolipids, monosialotetrahexosylganglioside (GM1) and monosialodihexosylganglioside (GM3), major components of the raft microdomain in the fibroblast plasma membrane, form cluster domains. When the cells were treated with methyl-β-cyclodextrin (MβCD) to extract free cholesterol, the degree of GM1 or GM3 clusterings significantly diminished^5^. These results indicate that GM1 or GM3 cluster domains depend on the presence of free cholesterol in the plasma membrane. However, the above treatments did not completely dissolve the clusters, suggesting that other mechanisms may be involved in the clustering of GM1 or GM3 in the cell membrane.

The invasion of erythrocytes by the malaria parasite *Plasmodium falciparum* is a complex, multistep process, and the sequence of invasive steps is probably similar for all *Plasmodium* species. In the first step of the invasion of the human erythrocyte, the merozoite attaches to the erythrocyte surface. This initial attachment is presumably mediated by the interaction between merozoite surface protein-1 (MSP-1) on the merozoite surface and band 3 in the erythrocyte plasma membrane^9^. *P. falciparum* MSP-1 is a GPI-anchored protein that is isolated in DRM fractions from schizont-stage parasites, an intraerythrocytic stage that consists of maturing merozoites enclosed in the parasitophorous vacuole (PV)^10^. In addition to MSP-1, other GPI-anchored merozoite surface proteins (MSPs), including MSP-2 and MSP-4, were identified in the DRM fractions of the parasite plasma membrane^10^. *P. falciparum* DRM-associated MSPs also contain six-cysteine (6-cys) family members that are considered to be involved in adhesion^10,11^. The erythrocyte plasma membrane also contains a small but complex set of proteins, which include band 3, CD59, Duffy antigen, stomatin, flotillin, and Gαs in rafts as DRM fractions^12,13^. Selective depletion of raft-cholesterol by treatment with MβCD dissociates all raft-associated proteins from DRM fractions, indicating that cholesterol is critical for all protein assembly into raft fractions. Of note, cholesterol depletion from the erythrocyte plasma membrane by MβCD can inhibit malarial invasion of the erythrocyte, although it does not have major effects on the shape, deformability, or transport properties of the erythrocyte^13^. It has also been shown that raft components are selectively internalized from the erythrocytes into the malarial vacuole^14^. Therefore, determining the localization of microdomains or raft components in the *P. falciparum* plasma membrane, PV membrane, and infected erythrocyte membrane at a nanometer scale would provide important insights into the localization of the raft-associated proteins and the biological processes involving rafts and their associated proteins.

Our results in this study demonstrated that GM3, a major component of the raft microdomain, was symmetrically localized in both the exoplasmic and cytoplasmic leaflets in the *P. falciparum* plasma membrane and PV membrane. This is the first time to show the localization of GM3 in the cytoplasmic leaflet of the eukaryotic organism membrane. Our QF-FRL is a useful method for the analysis of the topological and two-dimensional distribution of lipid molecules in the membranes of the *P. falciparum*-infected erythrocyte at the nanometer scale.

## Results

### Nanoscale-level distribution of GM3 on both the PF and the EF of the *P. falciparum* plasma membrane and the PV membrane

**Figure 1:**
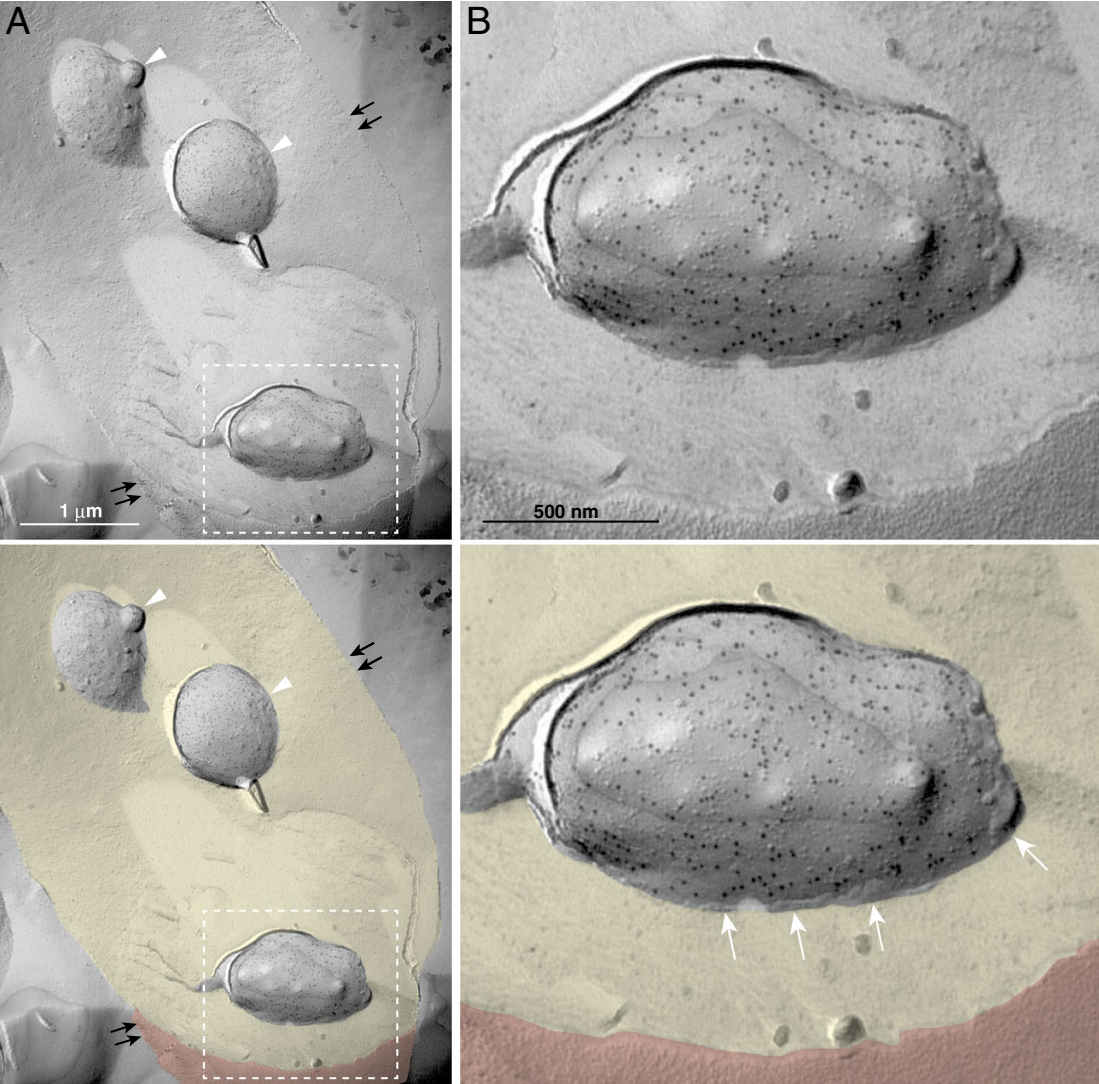
Observation of *P. falciparum* in a human erythrocyte. Using our freeze-fracture EM method, infected *P. falciparum* parasites were clearly observed inside human erythrocytes (white arrowheads). Double arrows indicate the erythrocyte plasma membrane. The labeling of anti-GM3 antibody was seen on the *P. falciparum* plasma membrane (A, B). The rectangle in A is magnified in panel B. In the lower panels of A and B, the plasma membrane and the cytoplasm region of *P. falciparum*-infected erythrocytes are colored with orange and yellow, respectively. White arrows indicate the luminal leaflet of the parasitophorous vacuole membrane. Scale bars: 1 μm (**A**) and 500 nm (**B**).

**Figure 2:**
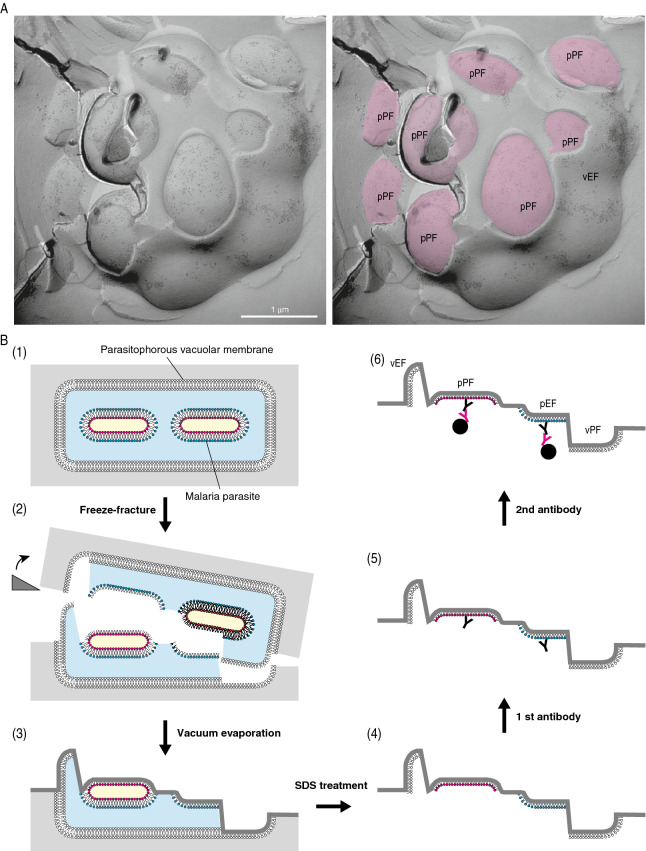
Outline of the QF-FRL method and GM3 localization in the membrane of live *P. falciparum*. (**A**) Freeze-fracture planes of *P. falciparum* at the schizonts stage consisting of maturing merozoites enclosed in a PV, comprising a single membrane bilayer. The P-faces are adjacent to the cytoplasm, and the E-faces to the exterior side. The labeling of anti-GM3 antibody was localized on both the *P. falciparum* EF (pEF) and *P. falciparum* PFs (pPF). In panel in A, pink represents the PF of the plasma membrane in *P. falciparum*. Scale bar: 1 μm. (**B**) Outline of the QF-FRL method in the *P. falciparum*-infected human erythrocyte. (1) QF: Live cells were quickly frozen without ice crystal formation. The metal contact freezing method was used in the present study. (2) Freeze-fracture: Frozen *P. falciparum* cells in human erythrocytes were fractured at below − 130 °C and under a high vacuum. Membranes were split into two leaflets, and the hydrophobic interface (i.e., the acyl chain side of the phospholipid monolayer) was exposed. (3) Vacuum evaporation: thin layers of carbon and platinum were deposited onto the hydrophobic interface of membranes to physically stabilize the molecules. Because platinum was evaporated at an oblique angle to the specimen’s surface (45°), protruding structures block the evaporating atoms to produce “shadows” behind the structures. Areas deficient in the platinum deposition, therefore, appeared to be electron-lucent under EM. Transmembrane proteins were seen as small bumps termed IMPs. (4) SDS treatment: Specimens were thawed and treated with an SDS solution to dissolve materials other than the lipid monolayer and integral membrane proteins, which were in direct contact with the carbon and platinum layer. This makes membrane proteins and lipid head groups accessible for antibody labeling (5). To visualize the antibody labeling under an electron microscopy, the first antibody was labeled with colloidal gold-conjugated secondary antibody on the replica specimens (6).

**Figure 3:**
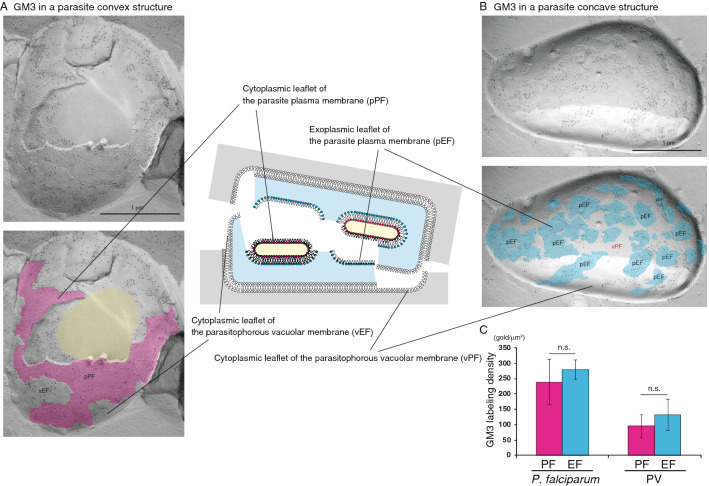
Raft component GM3 is localized in both the EF and the PF of the *P. falciparum* plasma membrane. In *P. falciparum*, the labeling of GM3 was detected on both the pPF (**A**, pink) and pEF (**B**, blue) leaflets of the plasma membrane. GM3 labeling was also observed on the PV membrane EF (vEF in **A**) and PV membrane PF (vPF in **B**). The cytoplasm of *P. falciparum* is colored with yellow (lower panel in **A**). Scale bars: 1 μm. (**C**) Labeling density of GM3 in the *P. falciparum* membrane and PV membrane in each leaflet. The labeling densities of GM3 on the PF (pink) and the EF (blue) of the *P. falciparum* plasma membrane and the PV membrane in the erythrocyte are shown. The gold labeling densities on the PF of the parasite plasma membrane and the PV membrane were comparable to those on the EF of each membrane. The mean ± s.e.m. of three independent experiments is shown. The labeling densities of GM3 on the PF were not significantly different (n.s., Student’s *t*-test) from those on the EF of both the *P. falciparum* plasma membrane and PV membrane.

After invading erythrocytes, most *P. falciparum* parasites develop to ring, trophozoite, then schizont stages, which contain newly produced daughter merozoites. Some parasites develop into male or female gametocytes. In our study, we observed mainly trophozoites and schizonts; gametocytes were rare (< 0.2% of all parasite structures in erythrocytes). Therefore, we examined asexual-stage parasites. *P. falciparum* can be clearly observed in erythrocytes using our freeze-fracture replica method (Fig. 1). Using high-resolution QF-FRL immunogold EM, we analyzed GM3 localization at the nanoscale level to precisely determine its distribution pattern in the *P. falciparum* plasma membrane in erythrocytes. In our previous study, we showed that gangliosides GM1 and GM3, major components of rafts, could be detected with anti-GM1 and anti-GM3 antibodies using thin-layer chromatography immunoblotting, dot blotting, and SDS-treated freeze-fracture replica immunogold EM^5,15^. We also showed that GM1 and GM3 antibody labeling was observed on the exoplasmic leaflet (E-face, EF), but not the cytoplasmic (protoplasmic) leaflet (P-face, PF), of the mouse fibroblast (MF) plasma membrane^5^. These results are consistent with the hypothesis that raft microdomains exist in the EF of the plasma membrane in mammalian cells^16^. Unexpectedly, the labeling of GM3 was strong on both the PF (Figs. 1B, and pink areas in 2A and 3C) and the EF (Fig. 3B, pEF, blue) in the plasma membrane of schizont-stage *P. falciparum*, which consists of maturing merozoites enclosed in the PV (Fig. 2A). The gold labeling density of GM3 on the PF was comparable to that on the EF of the *P. falciparum* plasma membrane (Fig. 3C). The freeze-fracture EM method showed that the PV membrane was detected as the smooth and intramembrane particles (IMPs)-deficient fractured face of both the EF and PF (Figs. 2 and 3). Interestingly, the GM3 labeling was also detected on both the PF and the EF of the PV membrane (Fig. 3). The gold labeling densities of GM3 of both sides of the PV membrane were much lower than those in the *P. falciparum* plasma membrane (Fig. 3C). These distribution patterns of GM3 in the parasite plasma membrane and the PV membrane were very similar among any parasites residing in the erythrocyte during the asexual developing stage.

In contrast, the same freeze-fracture EM analysis found no GM1 labeling on the PF or the EF of the *P. falciparum* plasma membrane and PV membrane (Fig. S1).

### PtdIns(4,5)P_2_ is localized in the PF, but not the EF, of the *P. falciparum* plasma membrane and the PV membrane

**Figure 4:**
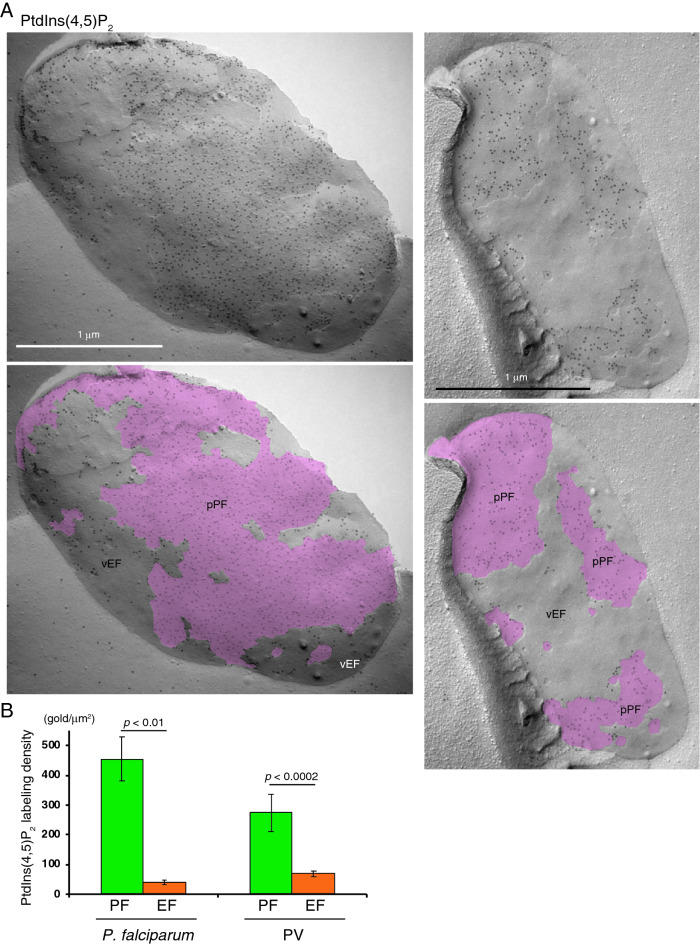
Localization of the PtdIns(4,5)P_2_ labeling on the PF of the *P. falciparum* plasma membrane and PV membrane. (**A**) The labeling of PtdIns(4,5)P_2_ was mainly observed on the PF in both the *P. falciparum* plasma membrane (pPF, pink) and PV membrane (vPF), although it was faintly identified on the EFs (vEF) of both membranes. Scale bars: 1 μm. (**B**) Labeling density of PtdIns(4,5)P_2_ in each leaflet of the *P. falciparum* plasma membrane and PV membrane. The labeling densities of PtdIns(4,5)P_2_ on the PF (green) and the EF (orange) of the *P. falciparum* plasma membrane and PV membrane are shown. The significantly higher gold labeling densities on the PF of both the *P. falciparum* plasma membrane and PV membrane were detected. Mean ± s.e.m. of three independent experiments is shown. The labeling densities of GM3 on the PF were significantly (Student’s *t*-test) and much higher than that on the EF of both the *P. falciparum* plasma membrane and PV membrane.

It is well known that in mammalian cells PtdIns(4,5)P_2_ is mainly localized in the plasma membrane and distributed on its PF, but not the EF, Previously, using the freeze-fracture EM method we demonstrated that the highly specific labeling of the probe GST-PLCδ1-PH, which selectively binds to PtdIns(4,5)P_2_, was detected on the PF, but not the EF, of the human fibroblast plasma membrane^17^. In the present study, we examined the distribution of PtdIns(4,5)P_2_ in the *P. falciparum* plasma membrane and the PV membrane using same QF-FRL method with the GST-PLCδ1-PH fusion protein as a probe. Gold particles for GST-PLCδ1-PH immunoreactivity were detected on the PF of the *P. falciparum* plasma membrane, indicating the presence of PtdIns(4,5)P_2_ (Fig. 4A). The average densities of gold particle PtdIns(4,5)P_2_ labeling on the PF and the EF of the *P. falciparum* plasma membrane were 454.4 ± 74.0 and 39.5 ± 8.5 particles/μm^2^ (mean ± s.e.m.), respectively (Fig. 4B). The gold labeling density of PtdIns(4,5)P_2_ on the PF of the *P. falciparum* plasma membrane was comparable to that of the human fibroblast plasma membrane (422.3 particles/μm^2^)^17^. In the PV membrane the PtdIns(4,5)P_2_ labeling was largely detected on the PF (Fig. 4). The gold labeling density of PtdIns(4,5)P_2_ on the PF of the PV membrane was significantly higher than that on the EF (Fig. 4B).

**Figure 5:**
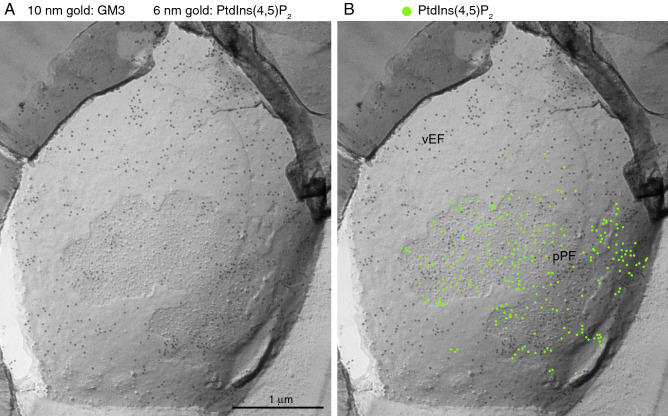
Double labeling of GM3 and PtdIns(4,5)P_2_ on the PF in the *P. falciparum* plasma membrane. The replica of the *P. falciparum* plasma membrane was stained simultaneously with anti-GM3 antibody and GST-PLCδ1-PH, which specifically binds to PtdIns(4,5)P_2_. Labeling of GM3 (10 nm gold) and PtdIns(4,5)P_2_ (6 nm gold, green dots in B) were colocalized on the PF in the *P. falciparum* plasma membrane. Scale bars: 1 μm.

To confirm the localization of GM3 on the PF in addition to the EF of the *P. falciparum* plasma membrane, we examined a replica of *P. falciparum* doubly labeled with the anti-GM3 antibody and the GST-PLCδ1-PH probe. The gold particles (10 nm) labeling of GM3 were colocalized with the labeling particles (6 nm, green) of PtdIns(4,5)P_2_ on the PF of the *P. falciparum* plasma membrane (Fig. 5), indicating that GM3 is on the PF of the plasma membrane.

### PtdIns(4,5)P_2_ is localized on the PF, but not the EF, of the erythrocyte plasma membrane

**Figure 6:**
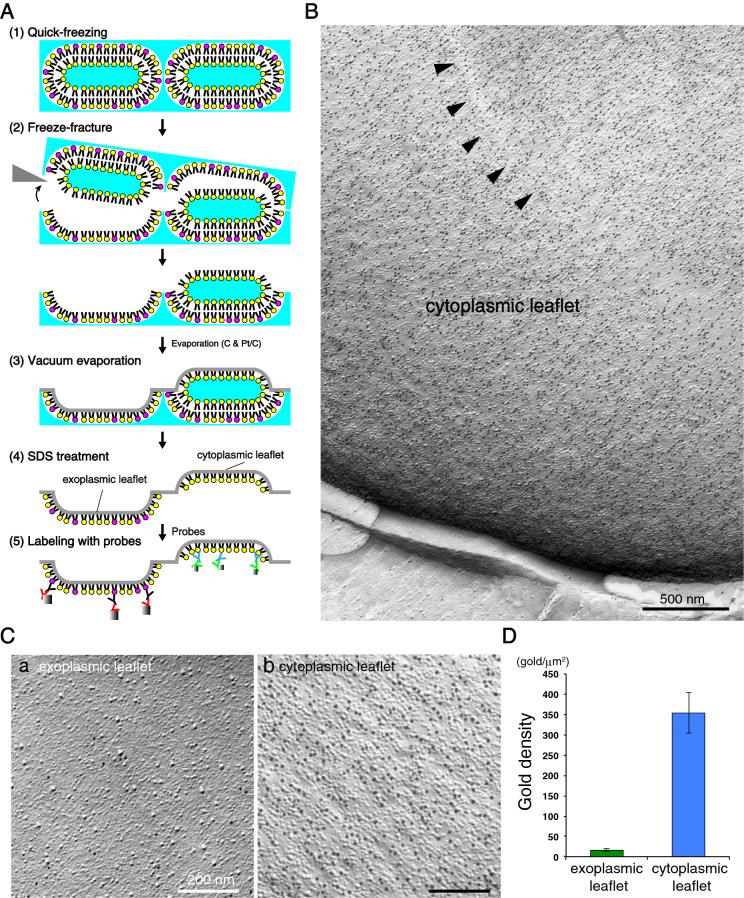
Localization of PtdIns(4,5)P_2_ in the PF, but not the EF, in the human erythrocyte plasma membrane. (**A**) Outline of a QF-FRL method for the uninfected erythrocyte. (**B**) A micrograph of the erythrocyte plasma membrane using a freeze-fracture EM method. Arrowheads represent the depressed area on the PF of the plasma membrane, which is characteristic of human erythrocytes^61,62^. The strong labeling of GST-PLCδ1-PH (PtdIns(4,5)P_2_) was detected on the PF of the erythrocyte plasma membrane. The labeling of PtdIns(4,5)P_2_ was localized on the PF (B, Cb), but not the EF (Ca), of the erythrocyte plasma membrane. (**D**) Labeling density of PtdIns(4,5)P_2_ in each leaflet of the erythrocyte plasma membrane. The labeling densities of PtdIns(4,5)P_2_ on the EF (green) and the PF (blue) of the erythrocyte plasma membrane are shown. Significantly higher gold labeling densities on the PF than that of the EF of the plasma membrane were detected. Mean ± s.e.m. of three independent experiments is shown. Scale bars: 500 nm (**B**) and 200 nm (**C**).

As expected, the labeling of PtdIns(4,5)P_2_ was observed on the PF, but not the EF, of the erythrocyte plasma membrane (Fig. 6). The gold labeling density of PtdIns(4,5)P_2_ on the PF of the erythrocyte plasma membrane was 353.7 ± 50.0 (mean ± s.e.m.). It is reasonable to assume that for most of the erythrocyte plasma membrane the labeling with GST-PLCδ1-PH reflects the real PtdIns(4,5)P_2_ distribution in the specimen, because approximately 50,000 PtdIns(4,5)P_2_ molecules per erythrocyte are estimated^18–20^.

*P. falciparum* readily invades mature healthy human erythrocytes, in which membrane invagination is uncommon, using the erythrocyte membrane to generate a host-derived PV membrane^21,22^. This is consistent with our result, in which PtdIns(4,5)P_2_ is localized in the PF, but not the EF, of both the PV and the erythrocyte plasma membranes (Figs. 4 and 6).

### Localization of GM3 of the uninfected and infected human erythrocyte plasma membrane

**Figure 7:**
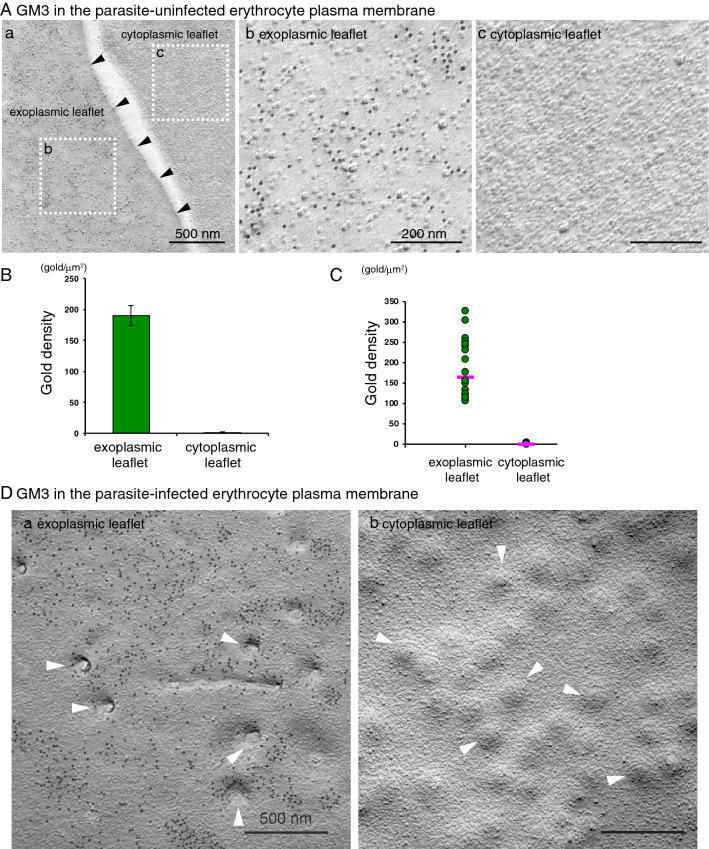
The expression of GM3 in the EF, but not the PF, of the uninfected and infected human erythrocyte plasma membrane. Freeze-fracture replicas of the erythrocyte were labeled by anti-GM3 antibody and colloidal gold (10 nm)-conjugated anti-mouse IgM antibody. (**A**) In the uninfected erythrocyte, GM3 labeling by 10 nm colloidal gold particles was detected in the EF of the freeze-fractured plasma membrane (**a**,**b**). The PF of the plasma membrane was unlabeled (**c**). The cell boundary is marked by arrowheads (**a**). The two rectangles in (**A**) are magnified in (**b**) (EF) and (**c)** (PF). The average (**B**) and scatter diagram (**C**) of gold labeling density of GM3 on both the PF and the EF of the plasma membrane showed a wide range for each sample on the EF. Pink lines in C indicate the median of each data. (**D**) In the infected erythrocyte plasma membrane, the knobs were clearly observed as indentations on the EF (arrowheads in **a**) or protrusions on the PF (arrowheads in **b**). As in the uninfected erythrocyte plasma membrane, the labeling of GM3 was detected on the EF (**a**), but not the PF (**b**) in the infected erythrocyte plasma membrane. Scale bars: 500 nm (**Aa**,**D**) and 200 nm (**Ab**,**Ac**).

It has been speculated that raft microdomains exist in the erythrocyte plasma membrane because it contains DRMs^12–14,23–25^. In this study, we examined the distribution of glycosphingolipids GM1 and GM3 in the erythrocyte plasma membrane using the QF-FRL immunogold EM technique. The labeling of GM3 was observed only on the EF, but not the PF, of the erythrocyte plasma membrane (Fig. 7). Immunogold labeling of GM3 showed clustered distribution (Fig. 7Ab). To analyze the complete gold patterns as rigorously and objectively as possible, we used well-established statistical methods for point pattern analysis. Twenty areas of 1 × 1 μm were randomly chosen from samples obtained from more than three independent experiments, and the distribution patterns were assessed by point pattern analysis using Ripley's K-function, which evaluates an exhaustive map of all interparticle distances over the study area and compares the observed distribution with that expected from complete spatial randomness (CSR)^26–28^. When data from all samples were compiled, the *L(r)—r* curve of the GM3 labeling did not show an evident peak (Fig. S2A), indicating that the size of the GM3 cluster was highly variable (Fig. S2B). The curve began to deviate from 99% confidence interval (CI) starting at a radius of 30 nm, meaning the radius of the GM3 cluster was generally larger than 30 nm. However, the gold distribution pattern of the GM3 labeling in the 20 chosen areas was variable (not shown). Also, the labeling density of GM3 on the EF was quite variable: 105 and 327 gold particles per μm^2^ in the lowest and highest density areas, respectively (Fig. 7C). The average density of the GM3 labeling in the replica was 189.5 gold particles per μm^2^ (Fig. 7B).

The infected erythrocyte plasma membrane surface has membrane protrusions known as knobs^29–31^. The freeze-fracture EM method showed that the knobs were indentations on the EF (arrowheads in Fig. 7Da) or protrusions (arrowheads in Fig. 7Db) on the PF of the erythrocyte plasma membrane when observed from the hydrophobic interface. The localization of GM3 in the plasma membrane of the infected erythrocyte was basically the same as that of the uninfected erythrocyte: the GM3 labeling was detected on the EF (Fig. 7Da), but not the PF (Fig. 7Db), of the infected erythrocyte plasma membrane.

In contrast to GM3, most labeling of GM1 was not observed on the EF of the erythrocyte plasma membrane when replicas were labeled with both anti-GM1 antibody (Fig. S3Aa) and b-ChTXB (Fig. S3Ab). This is different from our previous report for the MF, for which significant labeling of GM1 on the EF of the plasma membrane was detected (Fig. S3B)^5,15^. To validate this finding, we double labeled with anti-GM1 and anti-GM3 antibodies and found that both were detected in the MF plasma membrane (Fig. S3Bc), but only GM3 was seen in the EF of the erythrocyte plasma membrane (Fig. S3Ac). Ackerman et al.^32^ reported rare expression of GM1 in the erythrocyte plasma membrane by immuno-EM using ultrathin sections, which is consistent with our result, supporting that GM1 is rarely found in the EF of the erythrocyte plasma membrane.

## Discussion

The major findings of the present study are as follows: (1) the ganglioside GM3, which is a major component of the lipid raft, is abundantly localized in both the EF and the PF of the *P. falciparum* plasma membrane (Table S1); (2) GM3 is also localized in both the EF and the PF of the PV membrane; and (3) the phosphoinositide PtdIns(4,5)P_2_ is localized in the PF, but not the EF, of the *P. falciparum* plasma membrane and the PV membrane.

### Localization of raft microdomain component GM3 on both the EF and the PF of the *P. falciparum* plasma membrane and the PV membrane

The lipid composition of the two monolayers of the lipid bilayer in many biological membranes is strikingly different. It is widely accepted that glycosphingolipids are asymmetrically distributed in the EF, but not the PF, of the plasma membrane and the intracellular organelle membranes in eukaryotic cells^1,33^. Using the freeze-fracture EM method we showed in this and previous studies that glycosphingolipids GM1 and GM3, which are major components of the raft microdomain, are localized in the EF but not the PF of the plasma membrane of mammalian cells, including mouse fibroblasts and human erythrocytes (Fig. 7, Fig. S3)^5,15^. In this study, we found that this is not the case for *P. falciparum* plasma membrane and the PV membrane formed in the infected erythrocyte: GM3 is distributed symmetrically on both the EF and the PF of these membranes in similar amounts (Figs. 2, 3). Furthermore, we confirmed that GM3 is colocalized with PtdIns(4,5)P_2_, which is a well-known component of the PF of the biological membrane of mammalian and yeast cells (Fig. 5)^17,34,35^. This is the first study to demonstrate that a major component of the raft microdomain, GM3, is found in the PF of the biological membrane of eukaryotic cells.

Mature erythrocytes are terminally differentiated, devoid of all intracellular organelles, incapable of de novo protein or lipid synthesis, and lack endocytic machinery^36,37^. This means that invasion by *P. falciparum* merozoites into erythrocytes is complicated and that multiple processes and several invasive steps are involved. Invasion of the merozoite involves, (1) initial recognition of the erythrocyte surface receptor, (2) a reorientation process of apical end facing to the erythrocyte surface, (3) formation of a tight junction involving high-affinity ligand-receptor interactions, (4) the movement of a tight junction from the apical to the posterior pole powered by the merozoite’s actin-myosin motor, (5) proteolytic removal of the adhesive proteins at the junction upon reaching the posterior pole, (6) and a type of invasion that creates a PV using existing or newly synthesized proteins and lipids^38^. However, the basic and exact molecular mechanism involved in the attachment, reorientation, entry, and PV membrane formation by the parasite is still not clear. Some of the GPI-anchored proteins were localized on the surface plasma membrane of the *P. falciparum* merozoite, including MSP-1, MSP-2, MSP-4, and Pf12, were proposed to be functionally involved in erythrocyte invasion^9^. These GPI-anchored proteins were detected in DRM fractions isolated from schizont-stage *P. falciparum*, an intraerythrocytic stage that consists of maturing merozoites enclosed in the PV^10^. It was proposed that band 3, which is contained in the DRM fractions in the erythrocyte plasma membrane, was recognized by MSP-1^9,12^. Cholesterol depletion from the erythrocyte plasma membrane with MβCD, which results in the disruption of raft microdomains, inhibits the merozoite invasion of the erythrocyte^13^. This is consistent with the results in this study, in which GM3 was localized in the EF of the plasma membrane of both *P. falciparum* and the erythrocyte.

In the *P. falciparum* merozoite, the protein or proteins that link to the actin-myosin motor would be needed upon the merozoite invasion of the erythrocyte as described above. The motor complex that drives entry of the invasive Apicomplexan parasites has been studied most extensively in *Toxoplasma gondii*^38^, and the proteins involved appear to be highly conserved across these organisms, including *P. falciparum*^39^. In *T. gondii*, a glideosome, which is composed of myosin A heavy chain; myosin light chain; and two glideosome-associated proteins (GAPs), GAP45 and GAP50^40^, contains the molecular machinery for entry. This glideosome complex is consistently anchored to the inner membrane complex (IMC) via a transmembrane region in GAP50. However, the anchor of this glideosome machinery to the IMC can be broken by extracting cholesterol from the membrane via treatment with MβCD^41^. *P. falciparum* motility is also controlled by glideosomes. *P. falciparum* uses a complex similar to *T. gondii*, including PfGAP50, PfGAP45, myosin A, and two additional GAPs, PfGAPM1 and PfGAPM2, and it is also detergent-resistant^42^. Among components of the glideosome, MLC1 (MTIP) anchors to the IMC through its N-terminal palmitoylation^43^, GAP45 anchors to the PF of the parasite plasma membrane through N-terminal myristoylation and palmitoylation^44–46^, and at least two other IMC-related proteins, IMC1c and IMC1g, are palmitoylated^43^. Palmitoylation regulates the raft affinity of the protein^47^. Using the thin section EM method, Frenal et al.^46^ showed that lack of N-terminal acylation of GAP45 caused deformation and irregular spacing between the IMC and the plasma membrane in the parasite. They suggested that GAP45 is anchored to the plasma membrane by N-terminal acylation and to the IMC via its C-terminus, although there was no direct evidence showing that both GAP45 interactions occurred simultaneously. These results lead us to speculate that the raft microdomains suggested by GM3 localization in the PF of the *P. falciparum* plasma membrane in this study likely have important roles in glideosome activity via the interaction of GAP45 with the PF of the parasite plasma membrane. Further studies are needed to clarify the physiological role of the raft microdomains in the PF of the *P. falciparum* plasma membrane.

### The origin of the glycosphingolipid GM3 in the *P. falciparum* plasma membrane and PV membrane

Glycosphingolipid GM3 was detected in the *P. falciparum* plasma membrane (Figs. 2, 3). GM1 was not detected, however (Fig. S1). This is consistent with the results for the erythrocyte plasma membrane in which GM3, but not GM1, was localized in the EF (Fig. 7, Fig. S3). GM1 and GM3 are monosialic glycosphingolipids synthesized from glucosylceramide. The intraerythrocytic stage of *P. falciparum* includes an active glucosylceramide synthase that catalyzes the transfer of glucose to ceramide from UDP-glucose^48^. However, a previous report showed that *P. falciparum* did not biosynthesize or utilizes sialic acid^49^. These findings and the results of this study strongly suggest that GM3 is not synthesized in *P. falciparum*, but that the parasites obtain GM3 from the host erythrocyte. It has also been shown that raft components, including cholesterol, which is essential to maintain the raft microdomain structure^1^, are selectively incorporated to the PV membrane from the erythrocyte membrane upon *P. falciparum* invasion^14^. However, a nonspecific marker for the lipid bilayer (DiIC16) loaded to the erythrocyte membrane was not incorporated into the PV membrane upon parasite invasion, indicating that the molecular incorporation to the PV membrane must be highly selective for raft microdomain components^12,50^. This selective incorporation of raft microdomain components is important for the invasion process of *P. falciparum*, because depletion of cholesterol from the host erythrocyte plasma membrane results in incomplete parasite invasion into the erythrocyte^13^.

The membrane topologies of the raft microdomain components in the PV membrane of *P. falciparum*-infected erythrocytes are unclear. In this study, we found that a major raft component, GM3, which is usually distributed solely in the EF of the biological membrane, was detected in both the EF and PF of the PV membrane (Fig. 3). Haldar et al.^50^ demonstrated that an exogenously applied fluorescent phosphatidylcholine (PC) analog NBD-PC was incorporated to the PV membrane and parasite plasma membrane and suggested that the internalized NBD-PC was transported to the PV membrane and the parasite plasma membrane by monomer diffusion. However, it was not clear whether the transbilayer movement of NBD-PC occurred at the infected erythrocyte plasma membrane, because the back-extraction treatment mostly removed the exogenously applied NBD-PC from the infected and uninfected erythrocyte plasma membranes^50^. In this study, we found that GM3 was localized in the EF, but not the PF, in the *P. falciparum*-infected erythrocyte plasma membrane (Fig. 7D). We suggest that GM3 moves from the exoplasmic leaflet to the cytoplasmic leaflet by transbilayer lipid flip-flop at the PV membrane. However, the molecules responsible for the transbilayer flip-flop of ganglyosides such as GM1 or GM3 have not been identified^51,52^. However, some scramblases possess transbilayer moving activity for galactosylceramide and glucosylceramide in mammalian cells^53^ and for glycerophospholipids in *P. falciparum*^54^. Furthermore, in mammalian cells, it was reported that the phosphoethanolamine (PE) analog NBD-PE^55^ and phosphotidylserine (PS) analog NBD-PS^56^ were translocated from the plasma membrane to the intracellular organelles’ membranes by monomer diffusion when they are exogenously applied to the cell culture medium. Thus, it is plausible that GM3 localized in the PV membrane is diffused and moved to the parasite plasma membrane by monomer diffusion in a manner similar to that of NBD-PE and NBD-PS in mammalian cells. A transbilayer movement of GM3 from the PF to the EF of the PV membrane could replenish GM3 in the PF side of the PV membrane.

## Materials and methods

### Parasite lines and culture

The *P. falciparum* Dd2 parasite line was originally obtained from the United States’ National Institutes of Health. The parasites were maintained with O^+^ human red blood cells (RBC) at 2% hematocrit in fibrinogen-free human plasma-containing complete RPMI1640 medium. The use of human RBC and plasma was approved by the Ethics Committee, Institute of Tropical Medicine, Nagasaki University.

### Ethical approval

Human RBC and plasma for in vitro cultivation of *P. falciparum* were provided by Nagasaki Red Cross Blood Center. The usage of human RBC and plasma was approved by the Ethical Committee, Institute of Tropical Medicine, Nagasaki University. The experiment was conducted in accordance with approved protocols and regulations.

### Antibodies and probes

The recombinant glutathione-s-transferase (GST) fusion protein containing the phospholipase C (PLC)-δ1 PH domain (GST-PLCδ1-PH) was expressed in *Escherichia coli* and purified as described^17^. Rabbit anti-GM1 antibody was raised and affinity-purified as described^57^. The following antibodies and probes were purchased from commercial sources: mouse monoclonal (IgM) anti-GM3 antibody (clone GMR6; Tokyo Chemical Industry, Tokyo, Japan); biotin-conjugated cholera toxin B-subunit (b-CtxB; Invitrogen, Carlsbad, CA, USA); rabbit anti-GST antibody (Bethyl Laboratories, Montgomery, TX, USA); monoclonal mouse anti-biotin antibody (Jackson ImmunoResearch Laboratories, West Grove, PA, USA); 10 nm gold particle-conjugated anti-mouse IgG + IgM (EM.GAF10) and 10 nm gold particle-conjugated anti-rabbit IgG (EM.GAR10; BBI Solutions, Cardiff, UK); and 6 nm gold particle-conjugated goat anti-rabbit IgG antibody (Jackson ImmunoResearch Laboratories, West Grove, PA, USA).

### Quick-freezing and freeze-fracture

For quantitative lipid labeling of the biological membranes of *P. falciparum*-infected cells, uninfected normal human erythrocytes and mouse fibroblasts (MF) were quick-frozen using a metal sandwich quick-freezing method, quick-freezing and freeze-fracture labeling (QF-FRL). For metal sandwich freezing of *P. falciparum*, a small volume of the *P. falciparum*-infected erythrocyte pellet was placed on a copper foil and covered with a thin gold foil (~ 4 mm^2^ in area; 20 μm in thickness) and then frozen by a quick press between two gold-plated copper blocks precooled in liquid nitrogen^58,59^. For quick freezing of MF, cells grown on a small gold foil (~ 4 mm^2^ in area; 20 μm in thickness) were inverted on a prewarmed, thin layer of 10% gelatin on a copper foil with the cell side down and processed according to the metal sandwich method described above^58^.

The frozen specimens were transferred to the cold stage of a Balzers BAF400 apparatus (Bal-Tec AG, Lichtenstein) and fractured at − 130 °C under a vacuum of ~ 1 × 10^−4^ Pa. Replicas were produced by electron-beam evaporation in three steps: carbon (C; ~ 3 nm thick) at an angle of 90° to the specimen surface, platinum–carbon (Pt/C; 1–2 nm) at an angle of 45°, and C (10–20 nm) at an angle of 90°, as previously described (Fujita et al., 2010). The deposition thickness was adjusted by referring to a crystal thickness monitor (EM QSG100, Leica Microsystems, Wetzlar, Germany).

The thawed specimens were treated with 2.5% sodium dodecyl sulfate (SDS) in 0.1 M Tris–HCl (pH = 8.0) overnight at 60 °C–70 °C, and the replicas were stored in 50% buffered glycerol at − 30 °C until use.

### Labeling and electron microscopic imaging

Labeling with probes was performed as previously described^7,8,60^. Briefly, after rinsing, freeze-fracture replicas were blocked with PBS containing 3% bovine serum albumin (BSA) at room temperature for 30 min. Replicas were then incubated at 4 °C overnight with the following primary antibodies: anti-GM1 rabbit polyclonal antibody (1:50), anti-GM3 mouse monoclonal antibody (10 μg mL^−1^) or biotin-cholera toxin B-subunit (b-ChTXB) (10 μg mL^−1^) diluted in PBS containing 1% BSA. After washing with PBS containing 0.1% BSA four times, replicas were incubated at 37 °C for 30 min with 10 nm gold-conjugated secondary antibody in PBS containing 1% BSA for anti-GM1 or GM3 labeling, or with anti-biotin mouse monoclonal antibody followed by 10 nm gold-conjugated secondary antibody against b-ChTXB. For labeling PtdIns(4,5)P_2_, replicas were incubated with the GST-PLC-δ1-PH domain fusion protein (30 ng mL^−1^) in PBS containing 1% BSA at 4 °C overnight as previously described^17^. After washing with 0.1% BSA in PBS, the replicas were treated with rabbit anti-GST antibody (5 μg mL^−1^) for 30 min and then with 10 nm colloidal gold-conjugated anti-rabbit IgG antibody or 6 nm colloidal gold-conjugated donkey anti-rabbit IgG antibody (1:40) at 37˚C for 30 min. For the experiment illustrated in Fig. 2, double labeling of GM3 and PtdIns(4,5)P_2_ was performed. Briefly, replicas were treated with anti-GM3 antibody and GST-PLC-δ1-PH domain fusion protein (30 ng mL^−1^) at 4 °C overnight, and washed with PBS containing 0.1% BSA. Then, replicas were treated with rabbit anti-GST antibody and then colloidal gold (6 nm)-conjugated anti-rabbit IgG antibody and colloidal gold (10 nm)-conjugated anti-mouse IgG + IgM antibody at 37˚C for 30 min. Replicas were transferred to Formvar-coated grids and examined using a H7650 electron microscope (HITACHI, Tokyo, Japan) operated at 80 kV.

### Statistical analysis

EM images obtained from at least three independent experiments were used for the analyses. The number of colloidal gold particles was counted manually, and the areas were measured using ImageJ software (NIH). The labeling density in the selected structure was calculated by dividing the number of colloidal gold particles by the area. For each structure, the labeling density was measured in more than 10 different randomly captured micrographs. Statistical differences between the samples were analyzed using Student’s *t*-test.

## Supplementary Information

Supplementary Information 1.

Supplementary Information 2.

Supplementary Information 3.

Supplementary Information 4.

Supplementary Information 5.

## Abbreviations

b-ChTXB: Biotin-cholera toxin B-subunit
BSA: Bovine serum albumin
CI: Confidence interval
CSR: Complete spatial randomness
DRM: Detergent-resistant membrane
EF: Exoplasmic leaflet
EM: Electron microscopy
GAP: Glideosome-associated protein
GPI: Glycosylphosphatidylinositol
GM1: Monosialotetrahexosylganglioside
GM3: Monosialodihexosylganglioside
GST: Glutathione-s-transferase
GST-PLCδ1-PH: Glutathione-s-transferase (GST) fusion protein containing the phospholipase C (PLC)-δ1 PH domain
IMC: Inner membrane complex
IMP: Intramembrane particle
MβCD: Methyl-β-cyclodextrin
MF: Mouse fibroblast
MSP: Merozoite surface protein
MSP-1: Merozoite surface protein-1
NBD-PC: 1-Palmitoyl-2-[6[(7-nitro2-1,3-benzoxadiazole-4-yl)amino]caproyl]phosphatidylcholine
PBS: Phosphate buffer saline
PE: Phosphatidylethanolamine
PF: Cytoplasmic (protoplasmic) leaflet
PH: Pleckstrin homology domain
PLC: Phospholipase C
PS: Phosphatidylserine
PtdIns: Phosphatidylinositol
PV: Parasitophorous vacuole
QF: Quick-freezing
QF-FRL: Quick-freezing and freeze-fracture labeling
RBC: Red blood cell
SDS: Sodium dodecyl sulfate
