# Supplementary figures and images for: Glycosphingolipid GM3 is localized in both exoplasmic and cytoplasmic leaflets of Plasmodium falciparum malaria parasite plasma membrane
Source: Sci Rep. 2021 Jul 21;11:14890. doi: 10.1038/s41598-021-94037-3 (PMC8295280; doi:10.1038/s41598-021-94037-3)

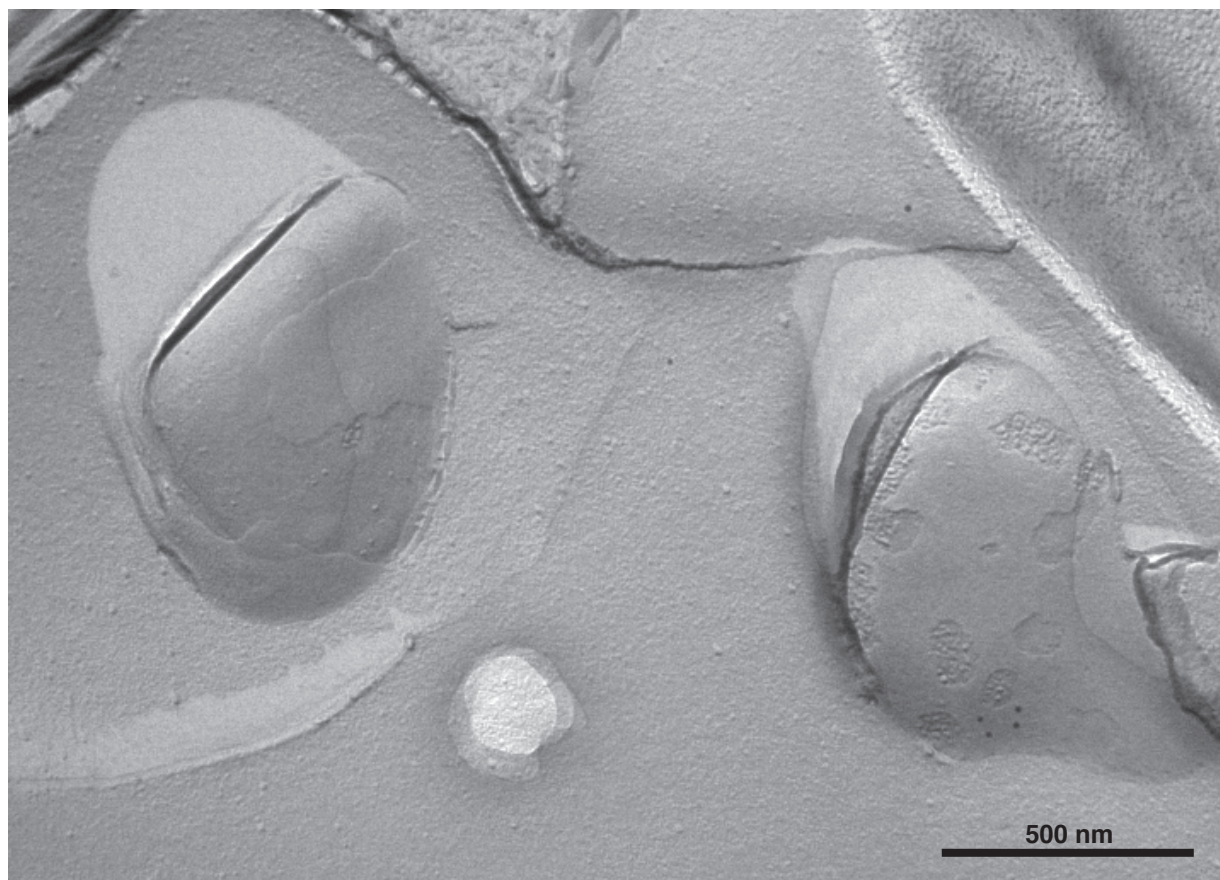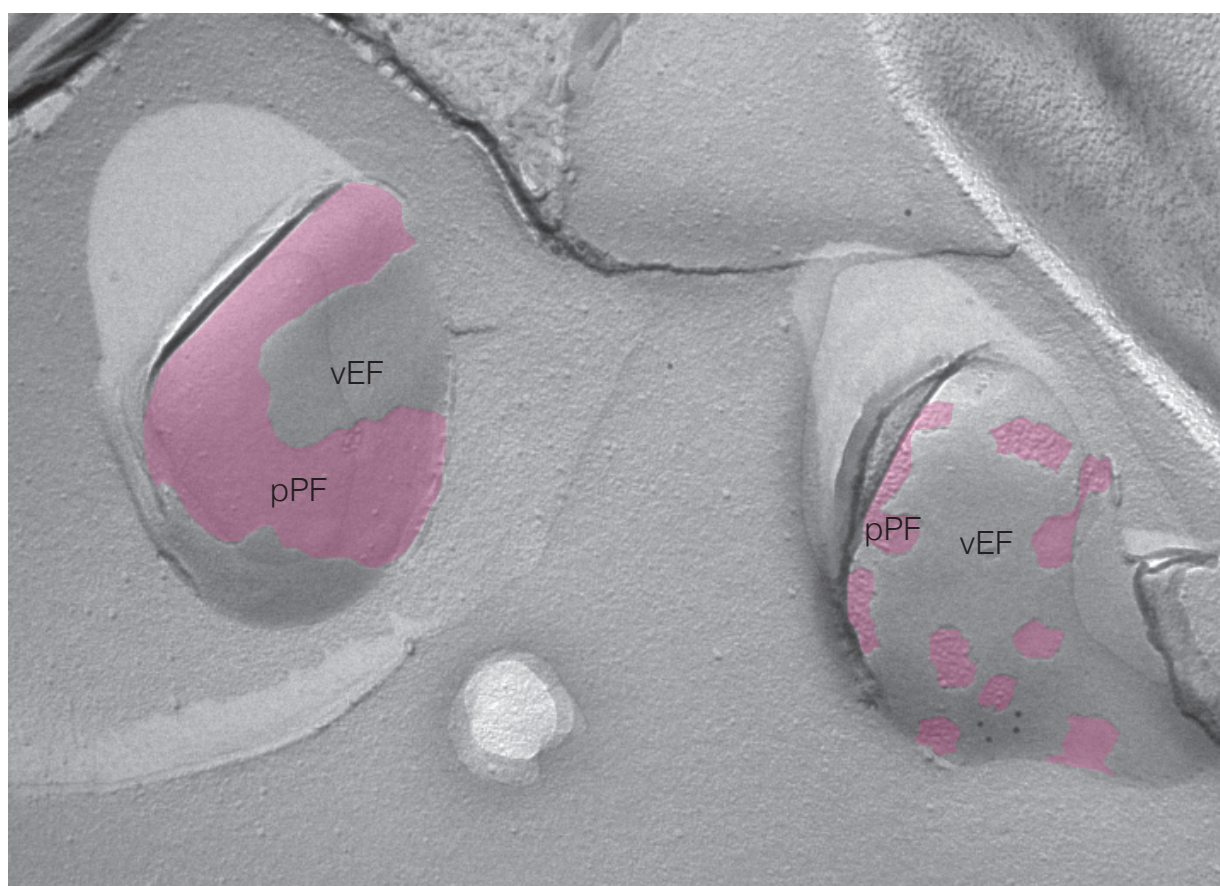

Fig. S1

Supplement: Supplementary file 1 — Supplementary Information 1. [file 41598_2021_94037_MOESM1_ESM.pdf]

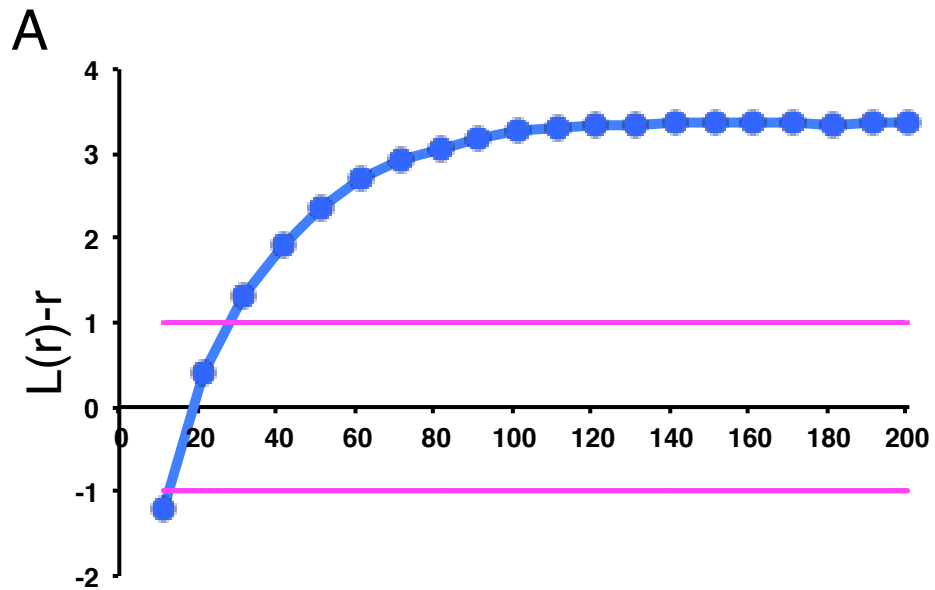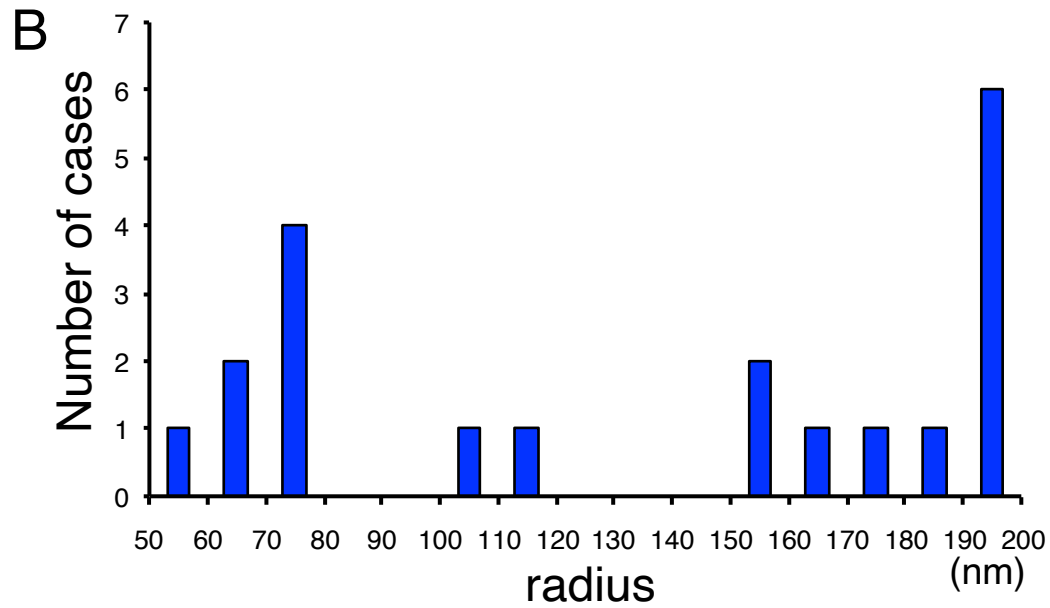

Fig. S2

Supplement: Supplementary file 2 — Supplementary Information 2. [file 41598_2021_94037_MOESM2_ESM.pdf]
