# Supplementary material for: Glycosphingolipid GM3 is localized in both exoplasmic and cytoplasmic leaflets of Plasmodium falciparum malaria parasite plasma membrane
Source: Sci Rep. 2021 Jul 21;11:14890. doi: 10.1038/s41598-021-94037-3 (PMC8295280; doi:10.1038/s41598-021-94037-3)
Supplement: Supplementary file 3 — Supplementary Information 3. [file 41598_2021_94037_MOESM3_ESM.pdf]

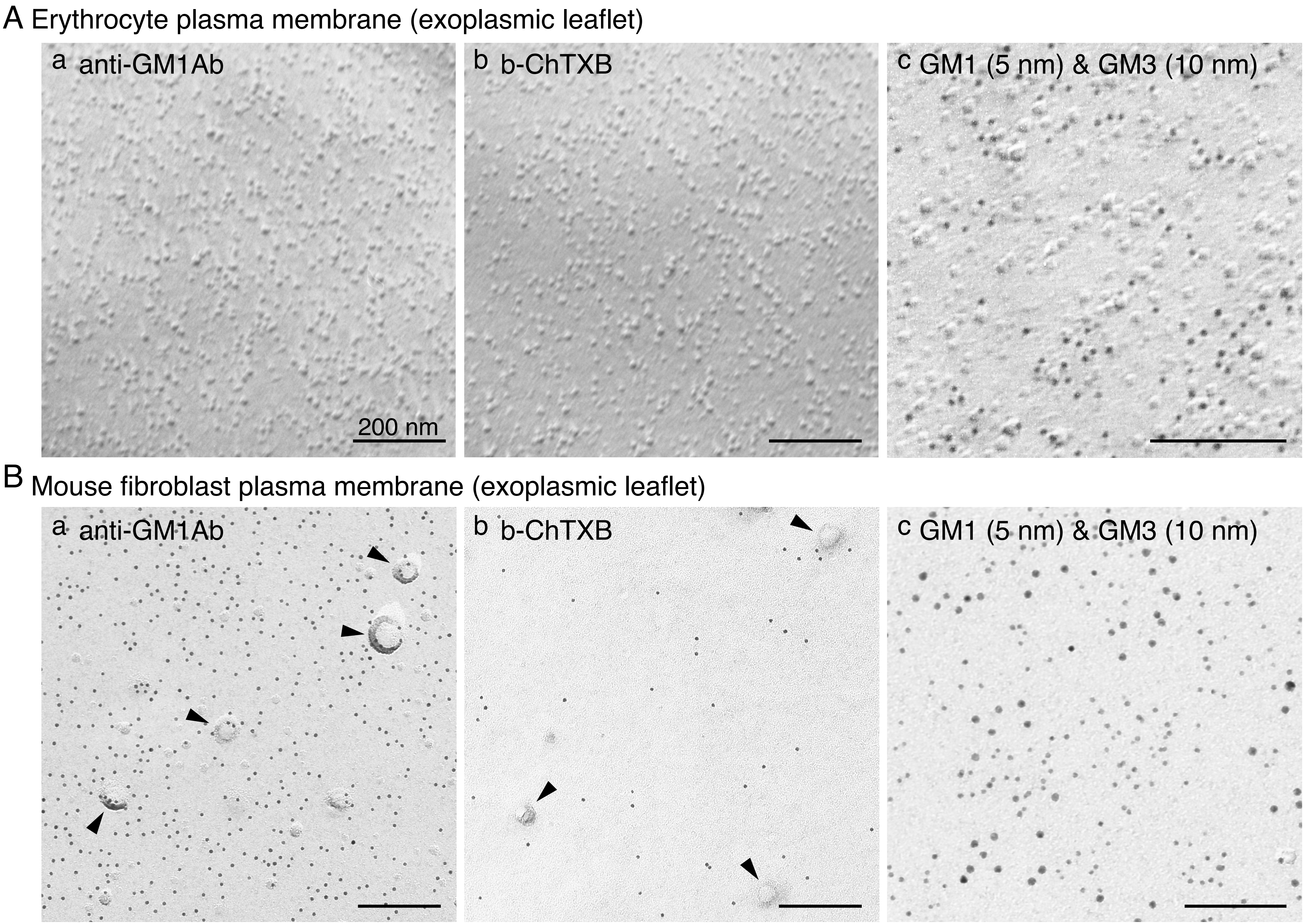
A Erythrocyte plasma membrane (exoplasmic leaflet)
a anti-GM1Ab
b b-ChTXB
c GM1 (5 nm) & GM3 (10 nm)
200 nm
B Mouse fibroblast plasma membrane (exoplasmic leaflet)
a anti-GM1Ab
b b-ChTXB
c GM1 (5 nm) & GM3 (10 nm)

Fig. S3
