# Supplementary material for: Glycosphingolipid GM3 is localized in both exoplasmic and cytoplasmic leaflets of Plasmodium falciparum malaria parasite plasma membrane
Source: Sci Rep. 2021 Jul 21;11:14890. doi: 10.1038/s41598-021-94037-3 (PMC8295280; doi:10.1038/s41598-021-94037-3)
Supplement: Supplementary file 4 — Supplementary Information 4. [file 41598_2021_94037_MOESM4_ESM.pdf]

		Gold density (gold/µm^2)	
		E-face (exoplasmic or luminal leaflet)	P-face (cytoplasmic leaflet)
**GM3**			
Plasmodium falciparum	plasma membrane	279.01 ± 32.31	238.56 ± 72.98
	parasitophorous vacuolar membrane	132.21 ± 49.64	95.05 ± 37.14
Infected erythrocyte	plasma membrane	191.17 ± 18.73	6.13 ± 4.45
Uninfected erythrocyte	plasma membrane	189.50 ± 16.47	1.56 ± 0.58
**PtdIns(4,5)P$_2$**			
Plasmodium falciparum	plasma membrane	39.50 ± 8.50	454.38 ± 73.97
	parasitophorous vacuolar membrane	69.51 ± 10.08	273.35 ± 64.09
Infected erythrocyte	plasma membrane	26.67 ± 4.72	217.0 ± 4.32
Uninfected erythrocyte	plasma membrane	16.00 ± 3.37	353.70 ± 49.96

Table S1. The average of gold labeling density on each fracture face of the *Plasmodium falciparum* plasma, parasitophorous vacuolar membranes, and the human erythrocyte plasma membrane.

The labeling densities of GM3 on the E-face were equivalent to the P-face of both the *P. falciparum* plasma membrane and parasitophorous vacuolar membranes, while the labeling density on the E-face was much higher than that on the P-face in the parasite-uninfected human erythrocyte plasma membrane. In contrast to GM3, in all *P. falciparum* plasma membrane, the PV membrane, and the human erythrocyte membrane, the labeling density of PtdIns(4,5)P$_2$ on the P-face was much higher than that on the E-face.
