# Supplementary material for: Glycosphingolipid GM3 is localized in both exoplasmic and cytoplasmic leaflets of Plasmodium falciparum malaria parasite plasma membrane
Source: Sci Rep. 2021 Jul 21;11:14890. doi: 10.1038/s41598-021-94037-3 (PMC8295280; doi:10.1038/s41598-021-94037-3)
Supplement: Supplementary file 5 — Supplementary Information 5. [file 41598_2021_94037_MOESM5_ESM.docx]

**Supplemental Figures**

**Fig. S1. No localization of GM1 in the plasma membrane of *P. falciparum* infected in the human erythrocyte.**

The freeze-fracture EM method found that GM1 labeling was not observed on the PF (pink) of the *P. falciparum* plasma membrane. Scale bars: 500 nm.

**Fig. S2. Objective analysis of the GM3 distribution pattern in the human erythrocyte plasma membrane.**

(A) Twenty areas (1 μm × 1 μm) were randomly photographed, and gold point patterns were analyzed by Ripley's K-function. The mean *L(r) - r* curve of GM3 did not show any apparent deflection peak from CSR (99% CI is shown by a pink line) below r = 200 nm. (B) Radii of maximal deflection for 20 sample areas ranging from 59 nm to 200 nm.

**Fig. S3. GM1 in the human erythrocyte plasma membrane.**

No labeling of anti-GM1 antibody (Aa) and biotin-cholera toxin B-subunit (b-ChTXB, Ab) was detected on the EF of the erythrocyte plasma membrane. In contrast, GM1 labeling was observed on the EF in the plasma membrane of MF using both anti-GM1 antibody (Ba) and b-ChTXB (Bb). Double labeling of GM1 (5 nm gold particles) and GM3 (10 nm gold particles) showed that both GM1 and GM3 are found in the EF of the mouse fibroblast (MF) plasma membrane (Bc). However, double labeling revealed that the labeling of GM3, but not GM1, was detected on the EF in the erythrocyte plasma membrane. Arrowheads in (B) show caveolae in the plasma membrane of MF. Scale bars: 200 nm.
